# Effect of training load on sleep parameters and biochemical fatigue markers in elite swimmers

**DOI:** 10.5114/biolsport.2023.124843

**Published:** 2023-06-26

**Authors:** Olga Surała, Jadwiga Malczewska-Lenczowska, Dariusz Sitkowski, Konrad Witek, Paweł Słomiński, Maciej Certa, Dawid Madej

**Affiliations:** 1 Institute of Sport – National Research Institute, Warsaw, Poland; 2 Józef Piłsudski University of Physical Education, Warsaw, Poland; 3 Warsaw University of Life Sciences – SGGW, Warsaw, Poland

**Keywords:** Sleep quality, Sleep quantity, Swimming, Endocrinology, Training load markers

## Abstract

The effect of strenuous exercise on sleep patterns in swimmers is equivocal. Therefore, the purpose of the study was to describe possible changes in sleep parameters among elite swimmers subjected to different training loads (TL). Methods: Eighteen elite swimmers (8 females) were monitored across two high-volume preparatory 1–wk periods (P1, P2) and a lower-volume tapering 1–wk period (P3) before a major competition. Internal (the session rating of perceived exertion [sRPE]) and external TL (training duration and volume) were measured, along with several sleep indices (e.g., bedtime, get-up time, sleep time, wake after sleep onset [WASO]). Serum measurements of urea, creatine kinase (CK), testosterone and cortisol were taken before and after training sessions at the beginning (Mondays) and end (Fridays) of each micro cycle. Athlete TL decreased significantly in a stepwise manner from P1 to P2 and from P2 to P3. Of all sleep parameters, only significant differences in bedtime and get-up time emerged (P3 > P1 and/or P2). Sleep duration (~6.3 h) or quality (WASO: 41–45 min) were also unaffected by TL. CK levels declined from P1 to P3 (d = -0.8), and from P2 to P3 (d = -0.6). Positive exercise-induced changes in CK were also seen in each training period. The other biomarkers did not show the same temporal or acute patterns. Irrespective of the TL, the monitored swimmers experienced insufficient and fragmented sleep across this study. Neither sleep quality nor quantity were affected by different magnitudes of TL. Among the biochemical markers of fatigue, baseline plasma CK activity best reflected the physiological response to TL.

## INTRODUCTION

Sleep is considered a principal component of training and competition recovery, and essential for optimizing athletic performance [1]. Given the importance of sleep to athletic performance and recovery, it is surprising that poor sleep is still prevalent among elite athletes [2]. Many factors can affect the sleeping patterns (e.g., travel, unfamiliar environment, pre-competition anxiety, the use of electronic devices, altitude) of athletic populations [2–5], including intensified training periods [2, 3]. For instance, a 3-wk overload training program promoted overreaching in triathletes and this, in turn, promoted a decline in sleep quantity and quality [6]. Similarly, a short period of intensified training affected sleep efficiency among male cyclists [7]. Others have, however, found no association between TL variation and sleep [8, 9]. These discrepancies could be explained by the interdependence of sleep-exercise interactions, such that sleep might drive, and be driven by, shifts in TL.

Other difficulties arise in terms of identifying the sleep component that best reflects TL. Taylor et al., [10] examined sleep changes in female swimmers during three phases of a competitive season. Most indicators of sleep quantity (e.g., total sleep time) and quality (e.g., sleep latency) did not differ across the season, but time spent in deep sleep was higher during the onset and peak training (vs. tapering) period. A more recent study profiled competitive swimmers across four training phases [9]. They found only trivial to small differences in sleep quantity between phases, whilst other parameters (e.g., sleep latency) showed only small to moderate differences. Substantial between-individual variation in sleep responses also emerged. Hence, a broad assessment of sleep, ideally combining subjective and objective measures, is needed to explicate more complex sleep-TL interactions. Adding to these difficulties, it is not clear whether sleep disturbances are a consequence of physical or psychological overload, thereby contributing to an overreaching or overtraining state [11], or simply a manifestation of the aforementioned syndromes.

Training load is generally classified into two components; internal and external [12]. Internal TL (e.g., heart rate response to training), reflects the psychophysiological response to exercise, whilst external TL (e.g., total distance covered), refers to the physical work completed. Both external TL [9, 13] and internal TL measures have been used in sleep research on athletes [14, 15], but few have combined these approaches to capture the nuances of sports training [16]. Others expanded their assessment to include salivary testosterone (T) and cortisol (C) measures [17, 18], but linkages to sleep were equivocal due to individual and context specificity, with both measures more strongly affected by psychological stress rather than fatigue [17, 18]. As such, implementing a panel of blood biomarkers could help decode the impact of training-related fatigue mechanisms on sleep patterns or vice versa.

Though none of the currently available blood biomarkers are regarded as the definitive fatigue indicator, some are considered promising and used in science and practice to monitor fatigue and the recovery process (e.g., creatine kinase [CK], urea [URE], T, C) [19, 20]. Serum CK activity, for example, reflects metabolic and/or mechanical damage of the muscle fibres resulting from repeated and intense contractions [21]. Increased serum URE is a marker of enhanced protein breakdown (in the absence of excessive protein intake) and stimulated gluconeogenesis that results from increased training loads [19, 20]. Both CK and URE may also provide information pertaining to increased muscular and/or metabolic strain [19, 20]. Cortisol is an important signal for stress and metabolic regulation, sometimes promoting catabolic processes, and synchronization of peripheral clocks with the circadian cycle [22]. Testosterone often works antagonistically to cortisol, promoting protein synthesis and reducing protein breakdown; thus, increased C and/or decreased T levels might indicate high physiological strain [20].

The purpose of this study was to describe possible changes in actigraphy-based and subjective sleep parameters among professional swimmers subjected to different TLs, assessed using a combination of external and internal TL measures. Specifically, the swimmers were monitored across three training micro-cycles that differed in prescribed load: two preparatory periods (P1, P2) with a relatively high TL and a pre-competition tapering period (P3) with a relatively low TL. We investigated the interplay between TL and sleep, and also whether any adaptive shifts would manifest as biochemical (URE, CK, T, C) changes, both baseline activity and acute exercise-induced reactivity. We broadly hypothesized that a higher TL in P1 and P2 would lead to a deterioration in sleep quality and/or quantity, along with changes in biochemical fatigue markers (e.g., increase in baseline and exercise changes in CK and C, lowering of baseline T), with restoration of these outcomes in P3 following a lower TL.

## MATERIALS AND METHODS

### Participants

Eighteen elite swimmers from one swimming team, subjected to one training plan, were recruited through convenience sampling. This cohort comprised of eight females (mean ± *SD*; height = 171.5 ± 4.5 cm, body mass = 64.7 ± 7.9 kg, training experience = 8 ± 2 years) and 10 males (mean ± *SD*; height = 184.5 ± 3.7 cm, body mass = 78.0 ± 3.6 kg, training experience = 13 ± 3 years) with a mean (± SD) age of 21 ± 4 years (range 16–28 years).These athletes competed at the highest national and/or international level with mean FINA points of 763 ± 110 (range 633 to 940 points). Inclusion criteria were age ≥ 16 years and swimming performance level of ≥ 600 World Aquatics points (formerly: Fédération Internationale de Natation, FINA points). Exclusion criteria were diagnosed sleeping disorder, tobacco use, use of sleep medications, travel across different time zones before the study commenced. All athletes were healthy and injury-free throughout the monitoring period. The participants were informed of the study aims, potential risks, procedures and benefits, after which they provided written informed consent. For participants under the consenting age of 18 years, informed consent was provided by their parents or legal guardians. This study received ethical approval (KEBN-19-43-OS) from the Ethics Committee of the Institute of Sport—National Research Institute, Poland, in agreement with the Helsinki Declaration.

### Study design

**FIG. 1:**
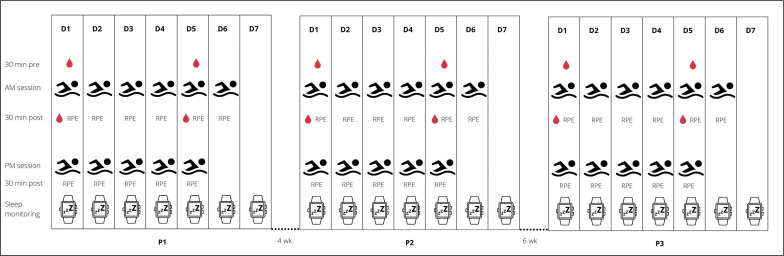
Schematic representation of the study design, including time points of training sessions, blood sampling, and the session rating of perceived exertion (sRPE) collection.

A single group within-subject design was used to address the study aim. The participants were monitored for weekly internal and external TL, sleep patterns, and biochemical changes across a 1-week block during three training periods (P1, P2, P3), as they prepared for the Polish National Swimming Championships. Four weeks of training separated P1 (aerobic endurance capacity; session duration = 113 ± 13 min, session volume = 6.4 ± 1.4 km) and P2 (aerobic endurance capacity; session duration = 111 ± 19 min, session volume = 5.3 ± 1.4 km), with six weeks separating P2 and P3 (tapering; session duration = 66 ± 9 min, session volume = 3.2 ± 0.4 km). P3 ended some three days before the championship event, where success was crucial to obtain a qualifying time standard for other international events (e.g., 18^th^ FINA World Championships, European Junior Swimming Championships). A traditional linear periodization plan (low-intensity and high-volume training across the first periods of the macrocycle, with progressive increases in training intensity and concurrent decreases in training volumes of the consecutive periods) was developed by the head trainer to ensure peak performance. To achieve this goal, an incremental decrease in training volume (in km) was prescribed from P1 to P2 to P3. Training frequency did not change within each micro cycle. This entailed 6 training days a week, comprising of 6 morning (starting 6:00 am in P1 and P2, 6:45 am in P3) and 5 afternoon (starting 4:00–6:00 pm) sessions. Figure 1 shows an overview of the study design as well as the collected parameters.

### Training load

Internal TL was measured using the session rating of perceived exertion (sRPE) method; a common approach in sport [23, 24] that offers a valid and easily accessible method for monitoring TL in competitive swimmers [23]. Each athlete provided a RPE (on a 0 to 10 scale) within 30 minutes of completing a swim-based training session. This score was multiplied by other metrics to derive two TL measures, as a function of: 1/training intensity and session duration expressed in min (sRPEh); 2/ training intensity and session distance expressed in km (sRPEkm). This dual approach was taken to account for a greater effect of training volume on perceived exertion than training duration in endurance sports like swimming [24]. The participants were familiarized with the RPE scale prior to study commencement. For P1, P2 and P3, weekly TL was calculated as the sum of all swim-based sessions completed during that micro cycle. Training load did not include non-specific training (i.e., gym sessions). Athlete training intensity was determined by a pre-study assessment of blood lactate concentration. Training intensity zones were classified as zone 1 (< 1 mmol/l; skills), zone 2 (1–3 mmol/l; aerobic capacity), zone 3 (3–5 mmol/l; aerobic capacity – threshold), zone 4 (> 5 mmol/l; anaerobic capacity), zone 5 (sprint) [25]. The division of training intensity into five different zones was adopted arbitrarily by the coaching staff.

### Sleep assessment

Sleep was assessed using a wearable sensor (ActiGraph GT3X-BT, USA) and daily sleep diary. The sensor was worn continuously (on the non-dominant hand) across each training period, except during swimming sessions or taking a bath. Raw actigraphy data were sampled at 30 Hz and converted to 60-second epoch files for analysis with dedicated software (ActiGraph v6.13.4, USA). Sleep and non-sleep periods were scored with the Cole-Kripke Algorithm [26], which has been shown to be more accurate not only in adults but also in children [27]. The sleep diary (e.g., quality of sleep, time in bed) was completed within one hour of getting out of bed. Diary information was also used to identify, and adjust, any mis-specified sleeping events from sensing data. The following sleep measures were obtained: 1/bedtime (hrs) – time when the subject went to bed; 2/ get-up time (hrs) – time when the subject got out of bed; 3/ sleep latency (min) – the number of minutes between the in bedtime and the sleep onset time for a specific sleep period; 4/sleep efficiency (%) – the percentage of time in bed that was spent asleep; 5/total sleep time (TST) (min) – the total number of minutes scored as “asleep.” at night; 6/wake after sleep onset (WASO) (min) – the total number of minutes the subject was marked awake after sleep onset occurred; 7/fragmentation index or FI (%) – the percentage of one minute periods of sleep vs. all periods of sleep in the sleep period; 8/self-reported sleep latency (min) – participant’s self-rating of the period of time between bedtime and sleep onset time; 9/subjective sleep score (1-5) – subjects’ self-rating of sleep quality (1 = very poor sleep quality to 5 = very good sleep quality). For exploration purposes, we also collected information on other factors likely to affect sleep, including: caffeine and alcohol intake, medication usage, health, appetite, willingness to train, pain, napping, and stress levels.

### Assessment of blood biochemistry

Our selection of blood parameters was based on several factors: 1/ whether they offer physiological insight into the mechanisms underlying exercise-induced fatigue; 2/ scientific evidence of their utility in relation to muscle fatigue or damage and sleep; 3/ previous use by team coaches and trainers to fine-tune training prescription [20, 28, 29]. These parameters were measured in capillary blood, as it offers several advantages over venipuncture techniques (eg., quick, simple and less invasive), leading to better athlete compliance.

Capillary blood samples were collected (within 45 minutes after waking) 30 minutes before (6:00 am ± 30 min) and 30 minutes after training sessions (8:00 am ± 30 min) at the beginning (Mondays) and the end (Fridays) of each micro cycle. Blood collection was adjusted to the training schedule, as well as the routine of monitoring fatigue indicators adopted by the coaches. Fingertip blood samples (660 *μ*l) were placed into Na-heparinized tubes, centrifuged at 2000 g for 10 minutes, then cooled prior to analysis. The samples were tested within three hours of collection. Plasma CK activity (U/l) and URE concentrations (mmol/l) were determined by spectrophotometry (Cobas Integra 400 Roche analyzer, Switzerland) with kit reagents from the manufacturer. Samples for assayed for T and C concentrations (nmol/l) using enzyme-linked immunosorbent kits (DRG, Germany). Inter-assay variability was less than 7% for CK, 4% for URE, 10% for T, and 7% for C. All assays were conducted by a certified technician in an accredited laboratory (number AB946) at the Institute of Sport – National Research Institute.

### Statistical analyses

Data were analysed in the R programming environment (version 3.5.1) (R Core Team, 2013) using the lmerTest package (version 3.1-0) [30]. The lmerTest package extends the ‘lmerMod’ class of the lme4 to provide p values for linear mixed-effect models. Given the longitudinal study design and missingness in our dataset (sleep and biochemical measures ~7%, TL ~4%), a linear mixed-effect model was deemed appropriate. Fitting was performed with a restricted maximum likelihood approach using Satterthwaite’s approximation to estimate denominator degrees of freedom and obtain p values. Significance was set at an alpha level of p < 0.05.

To evaluate the impact of TL on sleep and biochemical fatigue, we examined each set of variables using a one-way (training period [3 levels]) repeated measures analysis of variance. The same approach was used to test the biochemical outcomes, both baseline values and exercise-induced responses (pre to post changes). The day effect (Monday and Friday) was aggregated within this model to better capture any phasic response rather than day-to-day variability. Each model was specified with a random intercept and included gender as a covariate. Where appropriate, post-hoc assessments were performed using Tukey post-hoc contrasts. Post-hoc trends (p < 0.1) are highlighted if the effect size is medium or large, as they may reflect changes of practical significance for elite athletes. Prior to analysis, all variables were natural log-transformed to normalize data distribution and reduce non-uniformity bias. Log-transformed data are presented as back-transformed values in the original units or as a percentage change. Cohen’s d was calculated as an effect size statistic, with mean differences reported as a small (0.2 to < 0.5), medium (0.5 to < 0.8), or large (0.8+) effect. Significance was set at an alpha level of p < 0.05.

## RESULTS

**FIG. 2:**
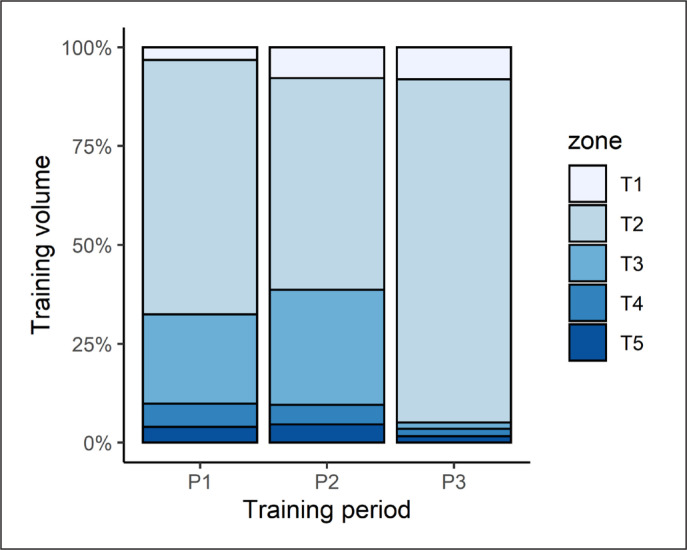
Percentage of training volume performed at a given intensity zone.

Athlete training intensity varied, based on five different “zones” of physical effort. The division of training intensity into five different zones was adopted arbitrarily by the coaching staff. The percentage of training volume spent in each zone is depicted in Figure 2.

### TL parameters

Our analyses yielded a significant (p < 0.001) effect of training period on all TL parameters (see Table 1). Post-hoc contrasts revealed that training duration (week) was shorter (p < 0.001) in P3 than both P1 (d = 4.3) and P2 (d = 4.1). Weekly training volume was highest in P1, before decreasing in P2 (d = -2.2, p < 0.001). A further reduction was observed in P3 that differed (p < 0.001) from P1 (d = -9.5) and P2 values (d = -7.7). Weekly TL (sRPEmin) was also significantly (p < 0.001) lower in P3 than that seen in P1 (d = -4.5) and P2 (d = 4.4). Similar patterns (all p ≤ 0.01) emerged for weekly TL (sRPEkm); being higher in P1 than P2 (d = 1.0) and P3 (d = 4.9), whilst P2 results also exceeded that of P3 (d = 4.1).

**TABLE 1:** Weekly training load in each training phase. Data are presented as estimated marginal means with a 95% CI.

Variables	Period 1 (P1)		Period 2 (P2)		Period 3 (P3)	
	Mean	95% CI	Mean	95% CI	Mean	95% CI
Training time (min)	1176	1086, 1261	1108	1022, 1188	620^AB^	572, 665
Training volume (km)	66.7	63.4, 70.8	54.6^A^	51.9, 57.4	28.2^AB^	26.8, 29.7
sRPEh (a.u.)	5219	4537, 5943	4770	4188, 5378	1604^AB^	1408, 1808
sRPEkm (a.u.)	299	260, 344	226^A^	198, 257	76.7^AB^	67.4, 87.5

A Significant from P1

B Significant from P2, all p < 0.05; a.u. – arbitrary units; sRPEh – session rating of perceived exertion as a function of training intensity and session duration expressed in minutes; sRPEkm – session rating of perceived exertion as a function training intensity and session distance expressed in km.

### Sleep parameters

The sleep parameters are shown in Table 2. Of all the measured sleep (quantity or quality) variables, only a significant training effect on bedtime (p = 0.013) and get-up time (p = 0.014) emerged. Contrast testing revealed a later bedtime in P3 compared with P1 (d = 0.9, p = 0.050) and P2 (d = 1.1, p = 0.017), and a later get-up time in P3 than P2 (d = 1.1, p = 0.021).

**TABLE 2:** Sleep quality and quantity measures in each training period. Data are presented as estimated marginal means with a 95%CI.

Variables	Period 1 (P1)		Period 2 (P2)		Period 3 (P3)	
	Mean	95% CI	Mean	95% CI	Mean	95% CI
Bedtime (hrs)	22:24	22:06, 22:42	22:24	22:06, 22:42	22:36^AB^	22:18, 22:54
Get-up time (hrs)	5:48	5:36, 6:00	5:30	5:24, 5:42	5:54^B^	5:42, 6:00
Total sleep time (min)	388	369, 403	376	358, 395	376	358, 395
Sleep efficiency (%)	88	87, 91	88	86, 90	88	86, 91
Sleep latency (min)	5	3, 6	4	3, 6	4	3, 5
SF sleep latency (min)	12	7, 17	11	8, 16	12	8, 16
Fragmentation index (%)	10	8, 12	11	9, 13	11	9, 14
WASO (min)	41	33, 51	45	36, 56	41	33, 51
SF sleep score (1-5 score)	3.6	3.3, 3.9	3.7	3.4, 4.0	3.7	3.4, 4.0

A Significant from P1

B Significant from P2, all p < 0.05; SF- self reported; WASO – wake after sleep onset

### Biochemical parameters

**FIG. 3:**
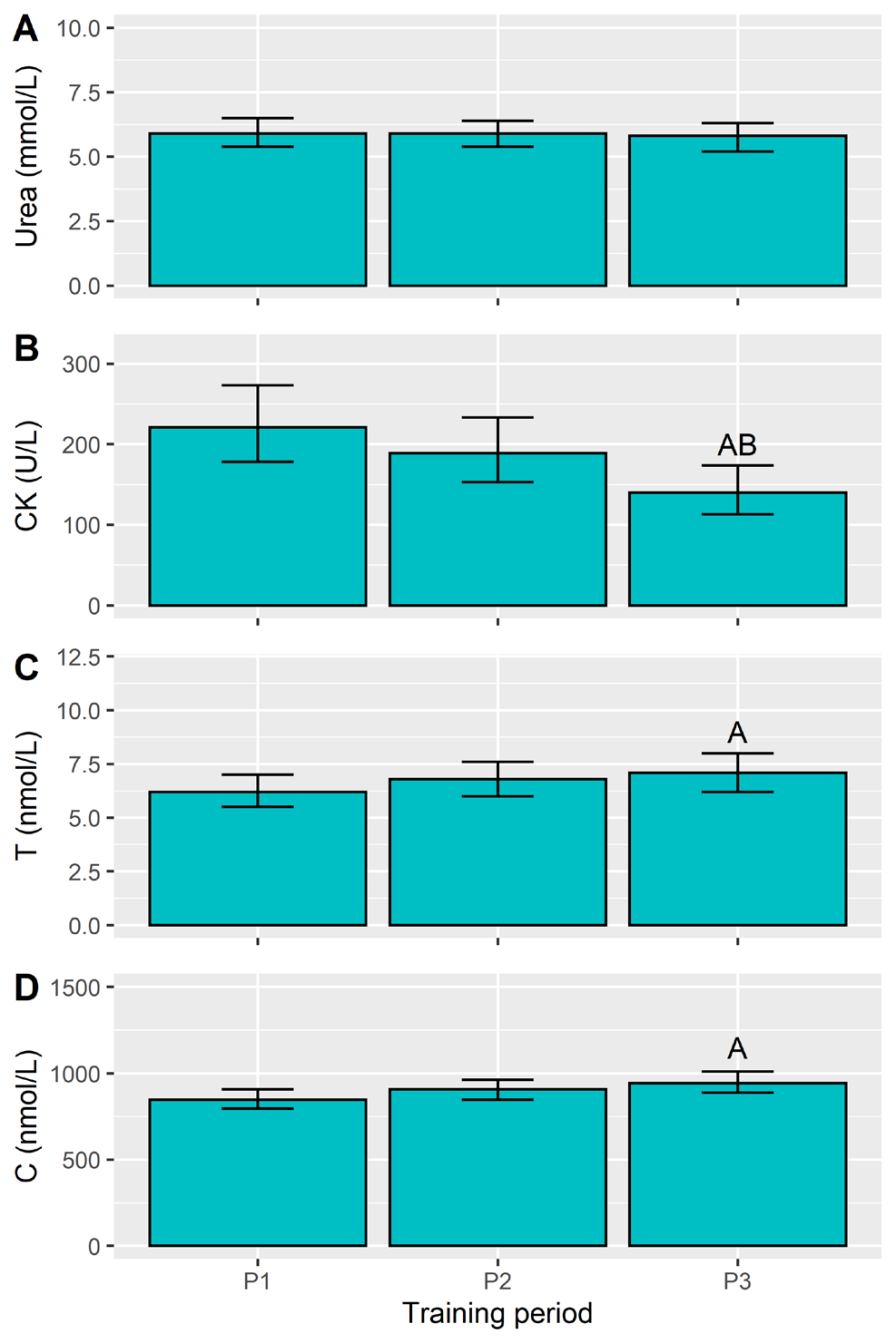
Pre-session urea, creatine kinase (CK), testosterone (T), and cortisol (C) concentration in each training period. Data are presented as estimated marginal means with a 95% CI. ASignificant from P1, BSignificant from P2, all p<0.05.

Training period did not affect (p = 0.682) pre-session URE concentration (Figure 3A). Pre-session CK (p = 0.001), C (p = 0.010) and T (p = 0.031) did, however, vary with respect to training phase. CK levels declined from P1 to P3 (Figure 3B) with a large effect (d = -0.8, p = 0.001), with a similar difference from P2 to P3 (d = -0.6, p = 0.036). Plasma T concentration rose from P1 to P3 (Figure 3C), a moderate effect (d = 0.7, p = 0.008), but neither differed from P2. A similar pattern emerged for C (Figure 3D), with a higher concentration in P3 than P1 (d = 0.6, p = 0.024), whereas P2 results were similar (non-significant) to both P1 and P3.

**FIG. 4:**
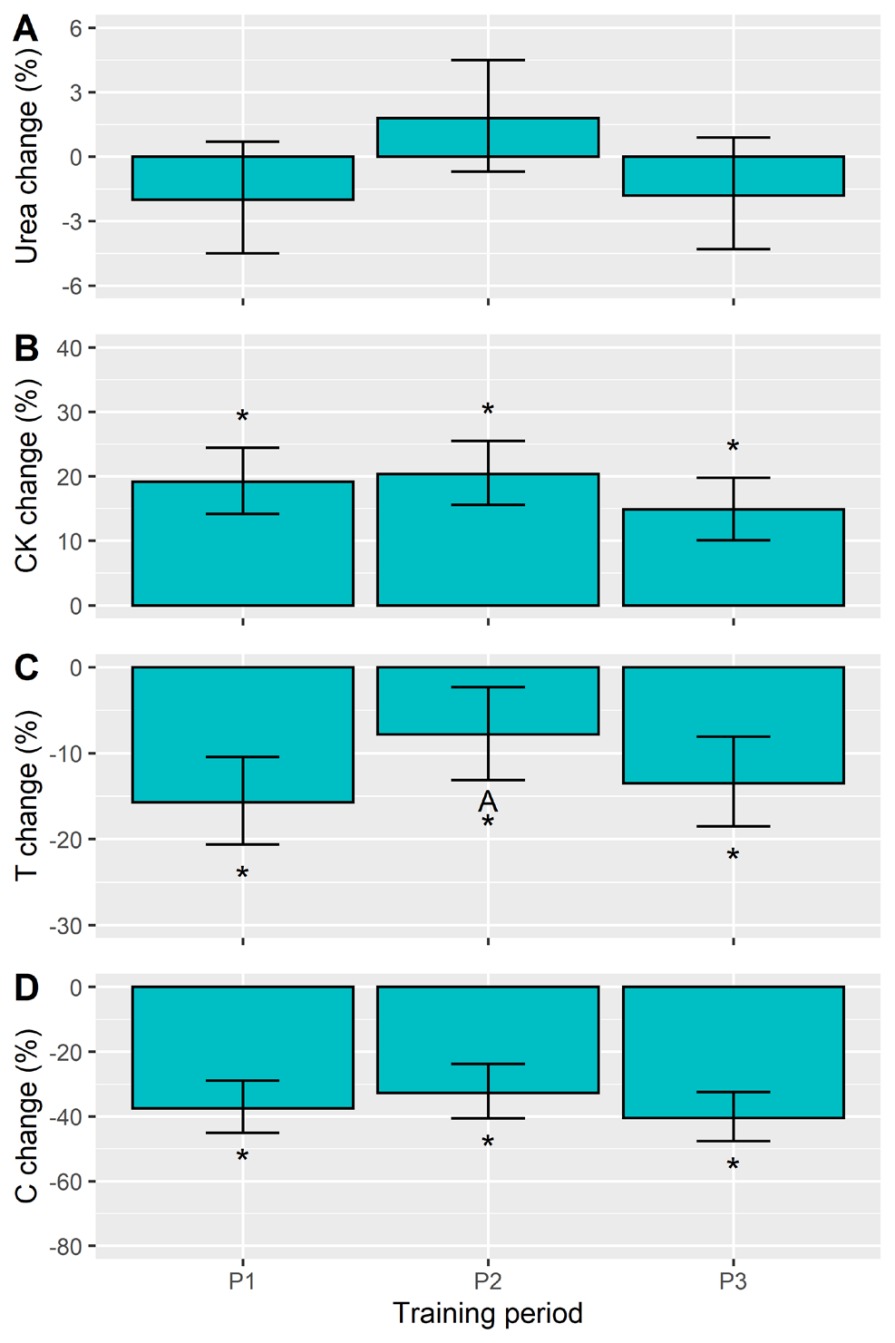
Acute within-session changes (%) in urea, creatine kinase (CK), testosterone (T), and cortisol (C) in each training period. The Monday and Friday sessions were pooled for the analysis. Data are presented as estimated marginal means with a 95% CI. *Significant from baseline, A Significant from P1, B Significant from P2, all p<0.05.

The within-session biochemical responses (%) were interpreted as being significant from baseline, if the 95% CI did not cross zero. We found no significant URE changes (Figure 4A), whereas positive CK changes were seen in each training period (Figure 4B). Conversely, a declining T (Figure 4C) and C (Figure 4D) response was observed in each period. When comparing these responses, a period effect on URE responsivity (p = 0.033) emerged, but the post-hoc comparisons did not reach statistical significance. The CK and C changes were unaffected by training phase (p ≥ 0.127). For T, the training effect was significant (p = 0.038) and follow-up tests identified a smaller (negative) change in P2 versus P1 (d = 0.6, p = 0.037).

## DISCUSSION

This study is the first to examine the interplay between TL, sleep and biochemical fatigue in elite swimmers across different training phases preceding a major competition. The swimmers experienced insufficient and fragmented sleep, regardless of the physical demands associated with different training periods. Only bedtime and get-up time differed (P3 > P1 and/or P2) in this study, but these differences resulted from the later start time of morning training across P3 (6:45) vs P1 (6:00) and P2 (6:00). Nevertheless, the magnitude of this difference was not large enough to be meaningful in real life; therefore, our hypothesis that higher levels of TL would affect sleep quantity and quality was not supported. Baseline plasma CK activity best reflected changes in TL, decreasing in a stepwise manner, although the anticipated rebound in P3 did not occur.

The impact of training load on sleep patterns in swimmers is equivocal [9, 10, 14, 15, 31]. As expected, all TL indices decreased incrementally over time with respect to P1, but with little corresponding change in athlete’s sleep quantity or quality. Only bedtime and get-up time differed in this work (P3 > P1 and/or P2), likely reflecting training schedule rather than reduced TL and shorter workouts prescribed during athlete tapering for the championship event. However, the results are not practically significant. Firstly, even though in P3 participants got out of bed later than in P1 and/or P2 they went to bed later as well. Secondly, changes in bedtime and get-up time did not translate to an increase in total sleep duration. A similar pattern was seen in swimmers during a 14–day altitude training camp at 1500 m [4]. In the monitored athletes, gradually increasing training loads (sRPE) did not jeopardize their sleeping patterns. Our findings also concur with with Walsh et al., [9] who reasoned that a threshold effect might be operating. That is, training differences in sleep parameters are more likely to occur when a much higher TL, and one that induces excessive overload, is prescribed. Still, there is no consensus as to whether impaired sleep is a cause or a consequence of heavy training loads [11, 32]. Sleep disturbances are common in sport, particularly among athletes experiencing training maladaptation that elicit overreaching or overtraining [6, 31]. Recently, Vachon et al. [32] reported that poor sleep quality prior to intensive training predisposed athletes to the development of functional overreaching. Throughout this study, all swimmers showed systematic improvements in performance, which indicates the prescribed TLs were not excessive, but entirely appropriate to promote positive adaptations. Moreover, based on the assessed fatigue biomarkers, it might be assumed that our subjects were not in an overreached or overtrained state.

Our major finding is that, irrespective of the training period and TLs, the swimmers slept less (~6.3 h) than the recommended 8–10 h or 7–9 h of sleep per night for healthy adolescents and adults, respectively [33, 34]. Supporting our results, many studies have reported disruptions in the sleep patterns of swimmers depending on their training schedule [9, 35–38]. For instance, the amount of sleep achieved by Australian swimmers during nights preceding training days (5.4 h) was substantially lower than prior to rest days (7.1 h) [35]. This reduction in sleep was linked to early morning training sessions (i.e., 6:00 am) and a failure to compensate with an earlier bedtime. Others have attributed low sleep quantity (i.e., 6.6 h) among swimmers to early-morning starts (i.e., 6:30 am) [36]. Correspondingly, a longer total sleep time (7.7–7.9 h) was found among swimmers who begun their first sessions at a later time of day (7:30–8:30 am) [9]. However, Vitale et. al. [15], who monitored sleep in swimmers 4 nights before and 14 nights during a 14-day training camp, frequently observed reduced sleep times (< 7 h) during this study, even though the athletes started training at 8:30 am. Thus, our observation of insufficient sleep quantity most likely stems from the early-morning workouts, scheduled around 6:00–6:45 am, which is typical of elite swimmers. The justification for this is to fit around schooling or work activities, and to provide maximum time for recovery between the sessions, since there is no performance rationale for early-morning starts. Interestingly, morning exercise might cause phase advances regardless of the chronotype [39]. People with late chronotypes, as often seen in young adults, are particularly susceptible to circadian disruption caused by misalignment of the internal rhythms with early-morning duties [39]. Further research is needed to confirm if swimmers with late chronotypes may benefit from phase advances induced by exercise in the morning. Although, participating in this study, being elites with several years of training experience, were training routinely in the early morning, so their sleep patterns were likely adjusted to the early morning start. Nonetheless, the negative impact of early-morning training sessions on sleep quantity is well recognized, as is the consequences of sleep restriction (e.g., fatigue, excessive daytime sleepiness, decreased exercise and cognitive performance, decreased mood) [2, 34].

Regardless of TL, the subjects did not meet recommendations for WASO (i.e., ≥ 41 min) [40], indicating restless or fragmented sleep. In the current study, swimmers spent less time awake after sleep was initiated (WASO: 41–45 min) than adolescent Icelandic swimmers (WASO : 64.8 ± 24.8 min) [38], but more than adult open-water swimmers (WASO: 22–27 min) monitored across altitude camp [15] or Dutch elite athletes (WASO: 33.3 ± 17.0 min) [8] from various sports disciplines during seven consecutive days of regular training. Impaired sleep continuity has been attributed to increased TLs [41], muscle soreness and general fatigue in athletes [14]. These data indicate that the swimmers obtained an insufficient amount of fragmented sleep, indicating an urgent need for sleep optimizing strategies focused on sleep extension and promoting continuous sleep. Hence, key swimming stakeholders should consider different strategies to maximize sleep, especially at the individual level. For instance, when feasible, delaying morning training sessions to allow longer sleep duration should be considered. Sleep hygiene education may encourage earlier bedtimes and reduce causes of excessive nighttime awakenings. Moreover, napping should be encouraged, particularly if extending nocturnal sleep is not possible.

To our knowledge, no previous research has investigated the impact of training on sleep in swimmers combining workload with blood fatigue biomarkers. Baseline plasma CK activity best reflected changes in TL, but with no corresponding change in athlete’s sleep pattern. Considering the entire preparation period, the most visible shifts in the biochemical parameters were identified for biochemical parameters prior to training sessions. This included a decline in CK levels, coupled with a gradual rise in T and C concentrations, as the athletes transitioned from high-volume training to low-volume, pre-event tapering. There are conflicting reports concerning fatigue biomarkers and TL in swimmers. In swimmers starting from second week of preseason training, CK levels have been shown to decrease across subsequent weeks of training [42] and tapering period [43–45]. Similar to our findings, T level decreased when swimming training intensified and increased during periods of decreased TL (i.e., post –taper) [44]. Even though the C increase across the taper period was unexpected, it was in line with previous findings as well [43]. On the contrary, some [46] have demonstrated weekly changes in URE but not CK, T or C levels in adolescent swimmers and triathletes, whilst others found no CK and endocrinological differences to periods of intense swimming training and taper [47].

Serum CK level is considered an indirect marker of muscle tissue damage [21]. Although swimming is a non-contact sport, where concentric muscle actions predominate over eccentric actions, increased TL and highly repetitive motions may enhance metabolic and/or mechanical disruption of muscle cells, leading to a rise in baseline serum CK changes [21], as we demonstrated herein. Also noteworthy is that the CK response to acute exercise was consistently elevated (by 15–20%) above baseline. Two possibilities could explain this finding: (1) exercise intensity was sufficiently high to induce changes in skeletal muscle membrane permeability, resulting in increased CK release independent of mechanically-induced damage; (2) swimming is a repetitive movement that places high demands on joint articulations and thus, leading to muscular strain that also promotes greater CK efflux [48].

The hormones T and C contribute to athletic performance and training adaption through several mechanisms [49]. One example being the anabolic to catabolic balance in muscle tissue regeneration and TL tolerance [50]. The observed rise in pre-training T level, when TL was the lowest, could represent greater pituitary activation following the more intense training period, leading to a downstream increase in T when tapering [50]. The corresponding increase in C might reflect a need to partition metabolic resources in preparation for the impending National Swimming Championships, where success was pivotal to obtain a qualifying time standard for 18th FINA World Championships and/or European Junior Swimming Championships [51]. In addition, it cannot be ruled out that higher C level might reflect an emotional response (eg. activation of the sympathetic nervous system and hypothalamic-pituitary-adrenal axis) in anticipation to a crucial sporting event [51]. We also observed a decline (by -8 to -40%) in T and C reactivity to exercise that arguably reflects normal circadian variation in steroid hormones [52], but no patterns emerged with regards to TL. Serum urea concentration (baseline and reactive) did not differentiate between high and low TLs, perhaps due to individual variability and other metabolic factors (e.g., availability of carbohydrates).

Finally, a number of important study limitations need to be considered. Although the biochemical parameters are recognized as surrogate markers of TL, their use as definitive biomarkers of fatigue should be done so with caution, namely due to high inter-subject variability and individual response patterns [46]. Furthermore, changes of plasma volume, that could affect post-training biochemistry, were not assessed. Another limitation is the lack of a chronotype assessment, which would allow for a better understanding of the impact of TL on sleep, since submaximal RPE and fatigue score are likely to be influenced by chronotype [53]. In addition, the sample size was small, due to recruitment restrictions when undertaking research on elite athletes, but this was offset somewhat by the repeated measures design and intensive monitoring procedures. Inferences of any TL effect are also limited by the number of assessment occasions, especially when the swimmers were already experiencing a high volume of training at study inception. Additionally, data were pooled across both sexes to increase our sample size. Recruiting a larger number of male and female swimmers would allow a more detailed examination of gender-related differences in the studied variables. Future studies should look to increase sample size, but where restrictions on recruitment are a limiting factor, it is advisable to adopt a repeated measures design to increase study power and subsequent inferences. Moreover, the phase assessment of TL did not include non-specific training (i.e., gym sessions), although the “dry-land” training presumably had no significant effect on the monitored parameters.

## CONCLUSIONS

In conclusion, the profiling of elite swimmers revealed insufficient and fragmented sleep, regardless of the physical demands associated with different training periods. In fact, neither sleep quality nor quantity were affected by different magnitudes of TL. Even though swimming is a sport with a predominant share of concentric muscle actions, baseline plasma (pre-training) CK activity best reflected the physiological response to TL among the monitored biochemical fatigue markers.

## References

[1] Vitale KC, Owens R, Hopkins SR, Malhotra A. Sleep Hygiene for Optimizing Recovery in Athletes: Review and Recommendations. Int J Sports Med. 2019; 40(8):535–43. doi:10.1055/a-0905-3103. 31288293 PMC6988893

[2] Gupta L, Morgan K, Gilchrist S. Does Elite Sport Degrade Sleep Quality? A Systematic Review. Sports Med. 2017; 47(7):1317–33. doi:10.1007/s40279-016-0650-6. 27900583 PMC5488138

[3] Nedelec M, Aloulou A, Duforez F, Meyer T, Dupont G. The Variability of Sleep Among Elite Athletes. Sport Med - open. 2018; 4(1):34. doi:10.1186/s40798-018-0151-2. PMC6063976 30054756

[4] Vitale JA, Galbiati A, De Giacomi G, Tornese D, Levendowski D, Ferini-Strambi L, Banfi G. Sleep Architecture in Response to a Late Evening Competition in Team-Sport Athletes. Int J Sports Physiol Perform. 2022; 17(4):569–75. doi:10.1123/ijspp.2021-0292. 35130508

[5] Vitale JA, Banfi G, Galbiati A, Ferini-Strambi L, La Torre A. Effect of a Night Game on Actigraphy-Based Sleep Quality and Perceived Recovery in Top-Level Volleyball Athletes. Int J Sports Physiol Perform. 2019; 14(2):265–9. doi:10.1123/ijspp.2018-0194. 30040006

[6] Hausswirth C, Louis J, Aubry A, Bonnet G, Duffield R, Le Meur Y. Evidence of disturbed sleep and increased illness in overreached endurance athletes. Med Sci Sports Exerc. 2014; 46(5):1036-45. doi:10.1249/MSS.0000000000000177. 24091995

[7] Killer SC, Svendsen IS, Jeukendrup AE, Gleeson M. Evidence of disturbed sleep and mood state in well-trained athletes during short-term intensified training with and without a high carbohydrate nutritional intervention. J Sports Sci. 2017; 35(14):1402–10. doi:10.1016/j.jsams.2017.07.003. 26406911

[8] Knufinke M, Nieuwenhuys A, Geurts SAE, Most EIS, Maase K, Moen MH, Coenen AML, Kompier MAJ. Train hard, sleep well? Perceived training load, sleep quantity and sleep stage distribution in elite level athletes. J Sci Med Sport. 2018; 21(4):427–32. doi:10.1016/j.jsams.2017.07.003. 28754605

[9] Walsh JA, Sanders D, Hamilton DL, Walshe I. Sleep Profiles of Elite Swimmers During Different Training Phases. J Strength Cond Res. 2019; 33(3):811–8. doi:10.1519/JSC.0000000000002866. 30289871

[10] Taylor SR, Rogers GG, Driver HS. Effects of training volume on sleep, psychological, and selected physiological profiles of elite female swimmers. Med Sci Sports Exerc. 1997; 29(5):688–93. doi:10.1097/00005768-199705000-00016. 9140908

[11] Lastella M, Vincent GE, Duffield R, Roach GD, Halson SL, Heales LJ, Sargent C. Can Sleep Be Used as an Indicator of Overreaching and Overtraining in Athletes? Front Physiol. 2018; 9:436. doi:10.3389/fphys.2018.00436. 29740346 PMC5928142

[12] Impellizzeri FM, Marcora SM, Coutts AJ. Internal and External Training Load: 15 Years On. Int J Sports Physiol Perform. 2019; 14(2):270–3. doi:10.1123/ijspp.2018-0935. 30614348

[13] Lalor BJ, Halson SL, Tran J, Kemp JG, Cormack SJ. A Complex Relationship: Sleep, External Training Load, and Well-Being in Elite Australian Footballers. Int J Sports Physiol Perform. 2020; 15(6):777-787. doi:10.1123/ijspp.2019-0061. 32023542

[14] Aloulou A, Duforez F, Léger D, De Larochelambert Q, Nedelec M. The Relationships Between Training Load, Type of Sport, and Sleep Among High-Level Adolescent Athletes. Int J Sports Physiol Perform. 2021; 16(6):890–9. doi:10.1123/ijspp.2020-0463. 33631716

[15] Vitale JA, Ieno C, Baldassarre R, Bonifazi M, Vitali F, La Torre A, Piacentini MF. The Impact of a 14-Day Altitude Training Camp on Olympic-Level Open-Water Swimmers’ Sleep. Int J Environ Res Public Health. 2022; 19(7):4253. doi:10.3390/ijerph19074253. 35409934 PMC8998594

[16] Thornton H, Delaney J, Duthie G, Dascombe B. Effects of Pre-Season Training on the Sleep Characteristics of Professional Rugby League Players. Int J Sports Physiol Perform. 2018; 13(2):176-182. doi:10.1123/ijspp.2017-0119. 28530487

[17] O’Donnell S, Bird S, Jacobson G, Driller M. Sleep and stress hormone responses to training and competition in elite female athletes. Eur J Sport Sci. 2018; 18(5):611–8. doi:10.1080/17461391.2018.1439535. 29482452

[18] Serpell BG, Horgan BG, Colomer CME, Field B, Halson SL, Cook CJ. Sleep and Salivary Testosterone and Cortisol During a Short Preseason Camp: A Study in Professional Rugby Union. Int J Sports Physiol Perform. 2019; 14(6):796–804. doi:10.1123/ijspp.2018-0600. 30569834

[19] Hecksteden A, Skorski S, Schwindling S, Hammes D, Pfeiffer M, Kellmann M, Ferrauti A, Meyer T. Blood-Borne Markers of Fatigue in Competitive Athletes - Results from Simulated Training Camps. PLoS One. 2016; 11(2):e0148810. doi:10.1371/journal.pone.0148810. 26891051 PMC4758695

[20] Urhausen A, Kindermann W. Diagnosis of overtraining: what tools do we have? Sports Med. 2002; 32(2):95–102. doi:10.2165/00007256-200232020-00002. 11817995

[21] Baird MF, Graham SM, Baker JS, Bickerstaff GF. Creatine-kinase- and exercise-related muscle damage implications for muscle performance and recovery. J Nutr Metab. 2012; 2012:960363. doi:10.1155/2012/960363. 22288008 PMC3263635

[22] O’Byrne NA, Yuen F, Butt WZ, Liu PY. Sleep and Circadian Regulation of Cortisol: A Short Review. Curr Opin Endocr Metab Res. 2021; 18:178-186. doi:10.1016/j.coemr.2021.03.011. 35128146 PMC8813037

[23] Wallace LK, Slattery KM, Coutts AJ. The ecological validity and application of the session-RPE method for quantifying training loads in swimming. J Strength Cond Res. 2009; 23(1):33–8. doi:10.1519/JSC.0b013e3181874512. 19002069

[24] Collette R, Kellmann M, Ferrauti A, Meyer T, Pfeiffer M. Relation Between Training Load and Recovery-Stress State in High-Performance Swimming. Front Physiol. 2018; 9:845. doi:10.3389/fphys.2018.00845. 30026704 PMC6041726

[25] Sozański H. Kierunki optymalizacji obciążeń treningowych. Wydaw. AWF [Akademii Wychowania Fizycznego]; 1992.

[26] Cole RJ, Kripke DF, Gruen W, Mullaney DJ, Gillin JC. Automatic sleep/wake identification from wrist activity. Sleep. 1992; 15(5):461–9. doi:10.1093/sleep/15.5.461. 1455130

[27] Quante M, Kaplan ER, Cailler M, Rueschman M, Wang R, Weng J, Taveras EM, Redline S. Actigraphy-based sleep estimation in adolescents and adults: a comparison with polysomnography using two scoring algorithms. Nat Sci Sleep. 2018; 10:13–20. doi:10.2147/NSS.S151085. 29403321 PMC5779275

[28] Urhausen A, Gabriel H, Kindermann W. Blood hormones as markers of training stress and overtraining. Sports Med. 1995; 20(4):251–76. doi:10.2165/00007256-199520040-00004. 8584849

[29] Buyse L, Decroix L, Timmermans N, Barbé K, Verrelst R, Meeusen R. Improving the Diagnosis of Nonfunctional Overreaching and Overtraining Syndrome. Med Sci Sports Exerc. 2019; 51(12):2524-2530. doi:10.1249/MSS.0000000000002084. 31274684

[30] Kuznetsova A, Brockhoff PB, Christensen RHB. lmerTest Package: Tests in Linear Mixed Effects Models. J Stat Softw. 2017; 82(13). doi:10.18637/jss.v082.i13.

[31] Wall SP, Mattacola CG, Swanik CB, Levenstein S. Sleep Efficiency and Overreaching in Swimmers. J Sport Rehabil. 2003; 12(1):1–12. doi:10.1123/jsr.12.1.1.

[32] Vachon A, Berryman N, Mujika I, Paquet J-B, Sauvet F, Bosquet L. Impact of tapering and proactive recovery on young elite rugby union players’ repeated high intensity effort ability. Biol Sport. 2022; 39(3):735–43. doi:10.5114/biolsport.2022.109453. 35959317 PMC9331337

[33] Hirshkowitz M, Whiton K, Albert SM, Alessi C, Bruni O, DonCarlos L, Hazen N, Herman J, Adams Hillard PJ, Katz ES, Kheirandish-Gozal L, Neubauer DN, O’Donnell AE, Ohayon M, Peever J, Rawding R, Sachdeva RC, Setters B, Vitiello M V, Ware JC. National Sleep Foundation’s updated sleep duration recommendations: final report. Sleep Heal. 2015; 1(4):233–43. doi:10.1016/j.sleh.2015.10.004. 29073398

[34] Paruthi S, Brooks LJ, D’Ambrosio C, Hall WA, Kotagal S, Lloyd RM, Malow BA, Maski K, Nichols C, Quan SF, Rosen CL, Troester MM, Wise MS. Consensus Statement of the American Academy of Sleep Medicine on the Recommended Amount of Sleep for Healthy Children: Methodology and Discussion. Clin Sleep Med. 2016; 12(11):1549-1561. doi:10.5664/jcsm.6288. PMC5078711 27707447

[35] Sargent C, Halson S, Roach GD. Sleep or swim? Early-morning training severely restricts the amount of sleep obtained by elite swimmers. Eur J Sport Sci. 2014; 14 Suppl 1:S310-5. doi:10.1080/17461391.2012.696711. 24444223

[36] Surda P, Putala M, Siarnik P, Walker A, De Rome K, Amin N, Sangha MS, Fokkens W. Sleep in elite swimmers: prevalence of sleepiness, obstructive sleep apnoea and poor sleep quality. BMJ open Sport Exerc Med. 2019; 5(1):e000673. doi:10.1136/bmjsem-2019-000673. PMC7010988 32095263

[37] Steenekamp T, Zaslona J, Gander P, Rowlands D, Leigh Signal T. Sleep/wake behaviour of competitive adolescent athletes in New Zealand: insight into the impact of early morning training. Sleep Med. 2021; 77:88–95. doi:10.1016/j.sleep.2020.11.023. 33341643

[38] Gudmundsdottir SL. Training Schedule and Sleep in Adolescent Swimmers. Pediatr Exerc Sci. 2020; 32(1):16-22. doi:10.1123/pes.2019-0067. 31592774

[39] Thomas JM, Kern PA, Bush HM, McQuerry KJ, Black WS, Clasey JL, Pendergast JS. Circadian rhythm phase shifts caused by timed exercise vary with chronotype. JCI insight. 2020; 5(3):e134270. doi:10.1172/jci.insight.134270. 31895695 PMC7098792

[40] Ohayon M, Wickwire EM, Hirshkowitz M, Albert SM, Avidan A, Daly FJ, Dauvilliers Y, Ferri R, Fung C, Gozal D, Hazen N, Krystal A, Lichstein K, Mallampalli M, Plazzi G, Rawding R, Scheer FA, Somers V, Vitiello M V. National Sleep Foundation’s sleep quality recommendations: first report. Sleep Heal. 2017; 3(1):6–19. doi:10.1016/j.sleh.2016.11.006. 28346153

[41] de Blasiis K, Joncheray H, Elefteriou J, Lesenne C, Nedelec M. Sleep-Wake Behavior in Elite Athletes: A Mixed-Method Approach. Front Psychol. 2021; 12:658427. doi:10.3389/fpsyg.2021.658427. 34413808 PMC8368439

[42] Rusnak M, VanderMeulen M, Byrd B, Byrd G, Rusnak R, Martin J, Hew-Butler T. Muscle Damage, Soreness, and Stress During Preseason Training in Collegiate Swimmers. Clin J Sport Med. 2021; 31(3):237-243. doi:10.1097/JSM.0000000000000736. 30870201

[43] Costill DL, Thomas R, Robergs RA, Pascoe D, Lambert C, Barr S, Fink WJ. Adaptations to swimming training: influence of training volume. Med Sci Sports Exerc. 1991; 23(3):371-7. 2020277

[44] Flynn MG, Pizza FX, Boone JBJ, Andres FF, Michaud TA, Rodriguez-Zayas JR. Indices of training stress during competitive running and swimming seasons. Int J Sports Med. 1994; 15(1):21-6. doi:10.1055/s-2007-1021014. 8163321

[45] Li Y, Zhu Y, Zhang J, Zhang X, Zeng Y. Biochemical changes and endocrine responses in pre-competition training in elite swimmers. Biol Sport. 2012; 29(1):71–5. doi:10.5604/20831862.984263.

[46] Julian R, Meyer T, Fullagar HHK, Skorski S, Pfeiffer M, Kellmann M, Ferrauti A, Hecksteden A. Individual Patterns in Blood-Borne Indicators of Fatigue-Trait or Chance. J Strength Cond Res. 2017; 31(3):608-619. 28212266 10.1519/JSC.0000000000001390

[47] Mujika I, Chatard JC, Padilla S, Guezennec CY, Geyssant A. Hormonal responses to training and its tapering off in competitive swimmers: relationships with performance. Eur J Appl Physiol Occup Physiol. 1996; 74(4):361-6. doi:10.1007/BF02226933. 8911829

[48] Brancaccio P, Maffulli N, Limongelli FM. Creatine kinase monitoring in sport medicine. Br Med Bull. 2007; 81–82(1):209–30. doi:10.1093/bmb/ldm014. 17569697

[49] Crewther BT, Cook C, Cardinale M, Weatherby RP, Lowe T. Two Emerging Concepts for Elite Athletes. Sport Med. 2011; 41(2):103–23. doi:10.2165/11539170-000000000-00000. 21244104

[50] Mujika I, Padilla S, Pyne D, Busso T. Physiological changes associated with the pre-event taper in athletes. Sports Med. 2004; 34(13):891–927. doi:10.2165/00007256-200434130-00003. 15487904

[51] van Paridon KN, Timmis MA, Nevison CM, Bristow M. The anticipatory stress response to sport competition; a systematic review with meta-analysis of cortisol reactivity. B BMJ Open Sport Exerc Med. 2017; 3(1):e000261. doi:10.1136/bmjsem-2017-000261. PMC5604718 29177073

[52] Guignard MM, Pesquies PC, Serrurier BD, Merino DB, Reinberg AE. Circadian rhythms in plasma levels of cortisol, dehydroepiandrosterone, delta 4-androstenedione, testosterone and dihydrotestosterone of healthy young men. Acta Endocrinol (Copenh). 1980; 94(4):536-45. doi:10.1530/acta.0.0940536. 6449126

[53] Vitale JA, Weydahl A. Chronotype, Physical Activity, and Sport Performance: A Systematic Review. Sport Med. 2017; 47(9):1859–68. doi:10.1007/s40279-017-0741-z. 28493061

